# No Learning Curve of the Direct Superior Approach in Total Hip Arthroplasty

**DOI:** 10.1111/os.12689

**Published:** 2020-05-18

**Authors:** Bouke J Duijnisveld, Joost A A M van den Hout, Robert Wagenmakers, Koen L M Koenraadt, Stefan B T Bolder

**Affiliations:** ^1^ Department of Orthopaedic Surgery Sint Maartenskliniek Nijmegen The Netherlands; ^2^ Department of Orthopaedic Surgery Amphia Hospital Breda The Netherlands; ^3^ Foundation for Orthopaedic Research Care and Education, Amphia Hospital Breda The Netherlands

**Keywords:** Direct superior approach, Learning curve, Mini posterior approach, Total hip arthroplasty

## Abstract

**Objectives:**

To assess the learning curve of the direct superior approach (DSA) for total hip arthroplasty (THA) and to compare surgical, clinical, and radiological results with a matched control group using the mini posterior approach (MPA).

**Methods:**

A prospective cohort study was performed from October 2016 to May 2017 including our first 52 patients undergoing THA using the DSA. Patients with primary osteoarthritis or osteonecrosis and a body mass index (BMI) < 35 who were eligible for surgery were included. As a control group, 52 patients who underwent the MPA were included, matched based on age, BMI, and ASA classification. In the DSA group, damage to the iliotibial tract and the distal external rotators, including the external obturator and quadriceps femoris muscles, was avoided. Outcome measures were collected, including surgical time, blood loss, postoperative pain, length of stay, implant position, use of walking aids, patient reported outcome measures (PROM), and complications. Unpaired *t*‐tests were used to analyze differences between the DSA and the MPA group in surgical time, blood loss, length of stay, and acetabular and femoral component position. χ^2^‐tests were used to analyze mobility and the number of complications. Two‐way repeated measures ANOVA was used to analyze pain scores and PROM between the DSA and the MPA groups.

**Results:**

The mean surgical time of 61 min (SD 8) in the DSA group was longer (*P* < 0.001) compared to that in the MPA group, 46 min (SD 12). No differences were found in blood loss, postoperative pain, or mean length of stay in the hospital. After 6 weeks, 94% of the patients in the DSA group were able to walk inside their home without walking aids compared to 90% in the MPA group. The mobility scores were not different after follow up of 6 weeks and 1 year (*P* = 0.12 and *P* = 0.36 respectively). All PROM improved postoperatively in both the DSA and the MPA group (*P* < 0.01). Acetabular cup and femoral stem position were not compromised by the DSA. Complications included two Vancouver B2 periprosthetic fractures in the DSA group, of which there was one surgical‐related fracture and one fracture after a traffic accident. Complications in the MPA group included one periprosthetic fracture, two hip dislocations, and one ischial neuropathy. No infections or thromboembolic events were observed. The 1‐year complication rate was not different between the MPA and DSA groups (*P* = 0.40).

**Conclusion:**

The DSA can be safely introduced as no learning curve in the prosthesis position or the complication rate was found.

## Introduction

Total hip arthroplasty (THA) is a highly successful operation to relieve pain and improve physical function in patients^1^. More than 1 million THA procedures are performed each year, and because of aging and obesity this number is still increasing^2^. The main indication for THA is advanced osteoarthritis of the hip. Other indications include avascular necrosis, post‐traumatic arthritis, rheumatoid arthritis, and developmental dysplasia of the hip. Cemented and uncemented implant designs have been developed and can provide excellent survival outcomes^3^. To improve early recovery, increasing attention is being paid to the approach to the hip to perform THA. The posterolateral approach is the most popular due to the excellent visibility it provides of both the acetabulum and the femur for both primary and revision hip arthroplasty^4^. The conventional posterolateral approach has evolved to the mini posterior approach (MPA) with good results^5^, ^6^, ^7^ and with decreased risk of hip dislocation since the introduction of 36‐mm femoral heads^8^. Other approaches include the anterolateral approach using the interval between the gluteus medius and the tensor fascia lata, and the direct lateral approach bisecting the gluteus medius fibers^4^. In the past decade, the direct anterior approach has become more popular due to faster recovery during the early postoperative period and improved gait compared to lateral approaches^9^, ^10^. However, increased risk of complications associated with the direct anterior approach have been discussed in the published literature, including femoral fractures, wound complications, neurovascular injury, and long‐term femoral stem loosening^8^, ^11^, ^12^, ^13^, ^14^. Furthermore, the direct anterior approach is difficult for surgeons to adopt and can be associated with a 20% complication rate during the long learning curve^14^, ^15^, ^16^, ^17^, ^18^. However, some surgeons have successfully introduced the direct anterior approach in their practice^19^, ^20^, ^21^. The functional benefit of one over the other surgical approaches in the long term is questionable, as previous studies have found no difference in hip function between the direct anterior, direct lateral, or posterolateral approach^22^, ^23^. The direct superior approach (DSA) is a new muscle‐sparing hip approach with similarities to the posterior hip approach. In addition to the MPA, the main objective of the DSA is to avoid damage to the iliotibial tract and the more distal external rotators^7^, ^24^, ^25^, ^26^. The DSA^25^, ^27^ is performed with the patient in the lateral decubitus position and the gluteus maximus muscle is split in line with its fibers with care not to incise the iliotibial band. The tendon of the piriformis muscle and the conjoined tendon are detached and upon closure reattached at their anatomical site. Angled reamers are used for the acetabulum to prevent damage to the iliotibial tract. To preserve the distal external rotators, preparation and component placement of the femur is performed with the leg in 40° of flexion, 40° of adduction, and 40° of internal rotation.

In most hip approaches the iliotibial tract is at least partially opened, which may be an important reason for postoperative pain in full weight standing and walking. If damage to the iliotibial tract can be avoided, less postoperative pain and earlier recovery may be expected. Retainment of the distal external rotators, together with an alternative capsulotomy with a direct side‐to‐side repair would increase hip stability and further decrease the risk of hip dislocation^26^. The introduction of any new surgical technique may require a learning curve, potentially worsening clinical results and increasing complication rates^28^. The learning curve of a new surgical technique should be investigated before starting large clinical studies exposing many patients to uncertain outcomes.

Recently, the surgical technique for the DSA was described^27^, and a cohort study^29^ and a review^30^ were published. However, no comparative studies have been conducted taking the learning curve of the DSA into account. Therefore, the objective of the present study was to compare the learning curve, surgical, clinical, radiological, and functional outcomes, and the complication rates between a prospective DSA cohort and a matched control group of MPA THA patients.

## Methods

### *Study design*

From October 2016 to May 2017 a prospective cohort study was performed including 52 patients as participants who were undergoing THA using the DSA. Inclusion criteria were: (i) patients with primary osteoarthritis or osteonecrosis; and (ii) patients with a body mass index (BMI) < 35. Exclusion criteria were: (i) patients with rheumatoid arthritis; (ii) patients with tumors; (iii) patients with serious hip deformity; and (iv) patients who had previous surgery to the hip or proximal femur. To measure the learning experience at the institute rather than single surgeon results, we included all patients who were operated on with the DSA. Two surgeons (JH and SB) were trained in the DSA technique through cadaver courses and surgeon to surgeon visits. Surgery was performed with a regular staff, who also followed theoretical and cadaver training together with the surgeons before starting to apply this technique with a clear protocol at our institute. The study was performed in compliance with the declaration of Helsinki. No ethical committee approval was needed as this study was purely observational.

The DSA cohort group was compared to a matched cohort of 52 patients who were operated on with the MPA in the period from January 2016 to May 2017 by the same surgeons. Patients were matched based on age, BMI, and American Society of Anaesthesiologists (ASA) classification using frequency matching^31^. No other changes were made in the surgical setup or in the postoperative care during the study period.

A Trident acetabular shell with an X3 polyethylene liner was used in combination with an Accolade TMZF stem and a 36‐mm head (Stryker Orthopedics, Mahwah, USA). In case of insufficient primary fixation of the uncemented trial shell, a cemented Rimfit cup was used (Stryker Orthopedics, Mahwah, USA). One patient in the DSA group and one patient in the MPA group received a 28‐mm head in combination with a small‐sized cemented cup. Age, gender, BMI, ASA classification, diagnosis, Charnley score^32^, and smoking habit were recorded.

### *Direct Superior Approach*

**Figure 1:**
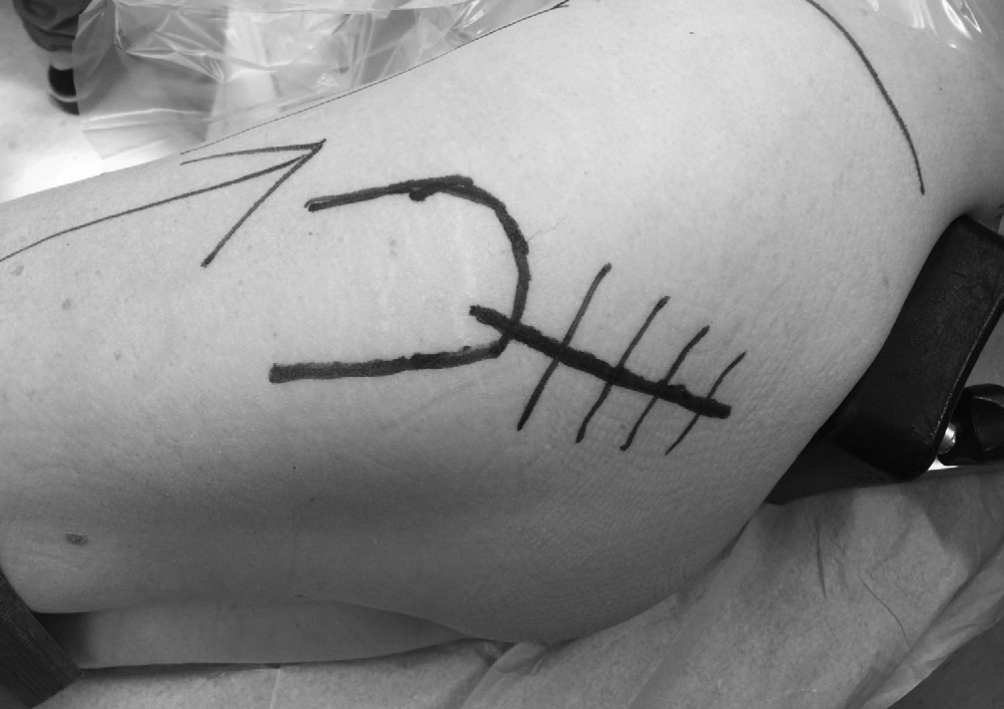
In the direct superior approach, an approximately 10‐cm incision is made from the posterosuperior corner of the greater trochanter, 45° to posterosuperior.

**Figure 2:**
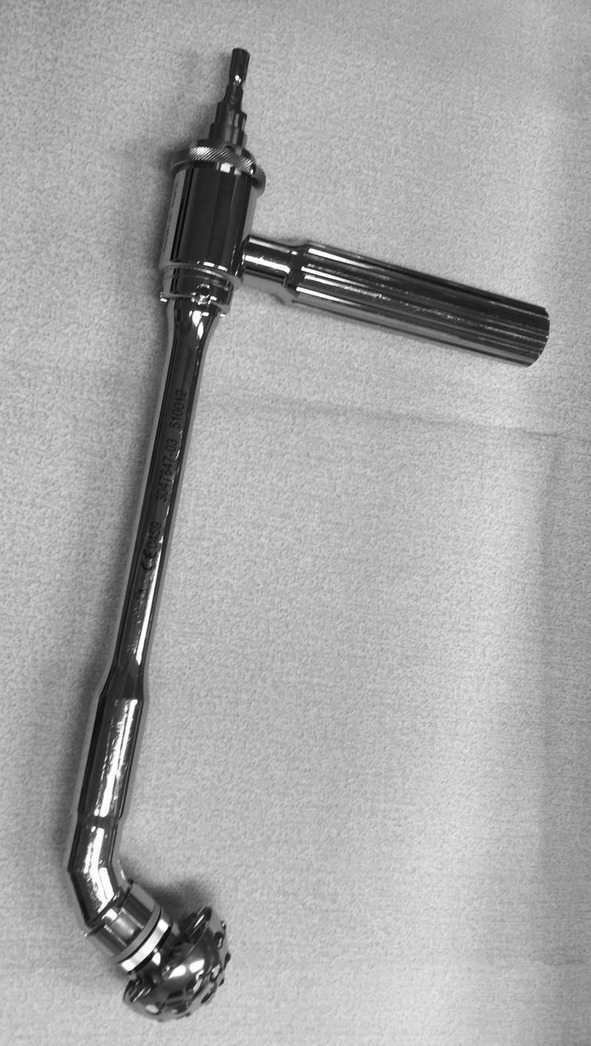
Angled reamers are used to prepare the acetabulum in the direct superior approach.

**Figure 3:**
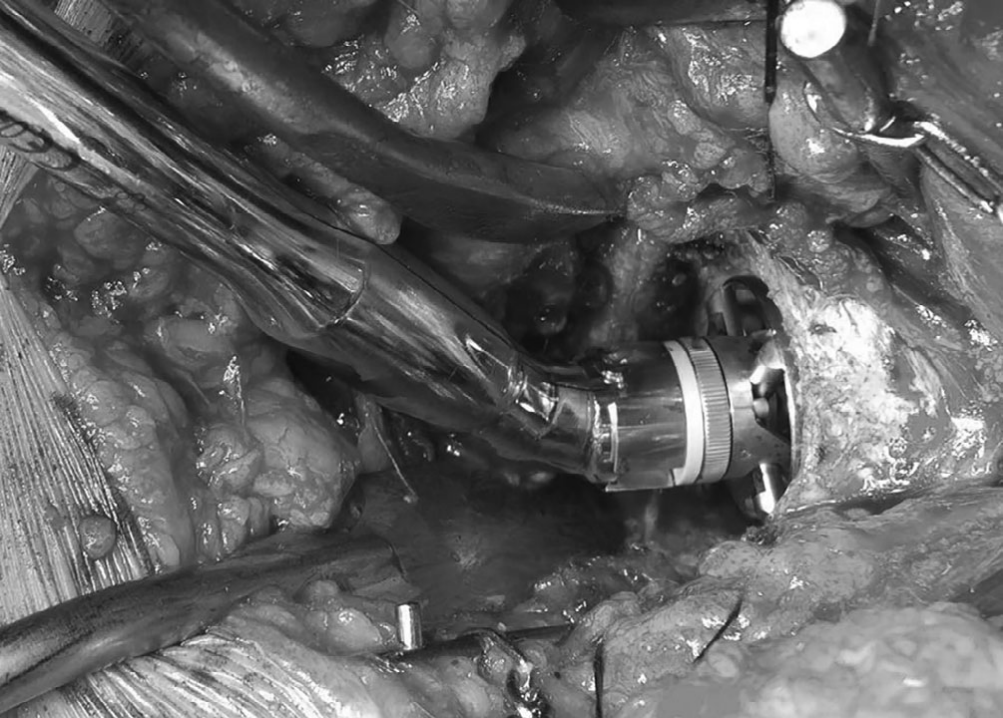
Angled reamer *in situ* during acetabulum preparation in the direct superior approach.

**Figure 4:**
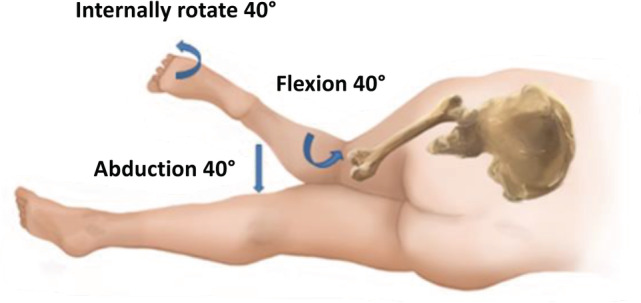
In the DSA, Femoral preparation and component placement is performed with the leg in 40° of flexion, 40° of adduction and 40° of internal rotation. Image reprinted with permission from Stryker Corporation. © 2017 Stryker Corporation. All rights reserved.

The DSA^25^, ^27^ was performed with the patient in the lateral decubitus position (Step 1). Starting at the posterosuperior corner of the greater trochanter, the incision was extended proximally for approximately 10 cm, angled 45° to the horizontal (Fig. 1, Step 2). The gluteus maximus muscle was split in line with its fibers (Step 3). At the distal end of the incision, care was taken not to incise the iliotibial band. The tendon of the piriformis muscle and the conjoined tendon were detached and reflected posteriorly (Step 4). The more distal external rotators, including the external obturator and the quadriceps femoris muscles, were left undetached. Superior capsulotomy^26^ was performed, from anterior distal to posterior proximal in line with the femoral neck (Step 5). After femoral neck resection (Step 6), angled reamers (Figs 2 and 3) were used to prepare the acetabulum and, subsequently, the acetabular component was placed (Step 7). Femoral preparation and component placement were performed with the leg in 40° of flexion, 40° of adduction, and 40° of internal rotation (Fig. 4, Step 8). At closure, direct side‐to‐side repair of the capsule (Step 9) and reattachment of the piriformis tendon and the conjoined tendon to their anatomical site were performed (Step 10).

### *Mini Posterior Approach*

The MPA was performed with the patient in the lateral decubitus position (Step 1). A 10–15‐cm incision was made over the posterior border of the greater trochanter (Step 2). Dissection was carried distally through the iliotibial band and proximally through the m. gluteus maximus (Step 3). The piriformis tendon and the conjoined tendon were detached and reflected posteriorly (Step 4). The capsulotomy was performed with a posterior capsular flap as close to the greater trochanter as possible to create a large capsular flap for reinsertion (Step 5). Femoral head resection was performed (Step 6). Straight reamers were used to prepare the acetabulum and the acetabular component was placed (Step 7). Femoral preparation and component placement were performed with the leg in 40° of flexion, 20° of adduction, and 90° of internal rotation (Step 8). Transmuscular or transosseous repair of the posterior capsule was performed depending on the preference of the surgeon, as published by Spaans *et al*.^5^ (Step 9). The conjoined and piriformis tendons were reattached to their anatomical site (Step 10).

### *Postoperative Care*

All patients were treated using the same standard rapid recovery protocol^33^. All patients received antibiotics for 24 h and antithrombotic prophylaxis for 6 weeks postoperatively. Patients were mobilized within 6 h after surgery, with direct full weight‐bearing allowed. During admission, all patients were instructed by a physical therapist. Patients were discharged home or transferred to an inpatient rehabilitation center based on their mobility and home support system. Discharge criteria included no or limited wound drainage, acceptable pain level, independent transfers in and out of bed, independent walking, and, if necessary, walking stairs. After discharge, patients continued rehabilitation with their own physical therapist and were allowed to mobilize without walking aids whenever this was possible.

### *Surgical Outcome*

Surgical time was defined as the time from skin incision until the time the skin was closed. Perioperative blood loss was measured, as well as the hemoglobin level preoperatively compared to the hemoglobin level in the morning of the first postoperative day.

### *Clinical Outcome*

Pain was measured using a numeric rating scale (NRS) scale from 0 to 10, with 0 indicating no pain and 10 maximal pain. The pain score was measured postoperatively on return to the orthopaedic ward and at discharge from the hospital. The length of stay (LOS) in the hospital was measured in days. Whether the patient was discharged to their home or to an inpatient rehabilitation center was recorded. Mobility was scored 6 weeks postoperatively at the outpatient clinic as: (i) use of walking aids inside their home as well as outside; (ii) use of walking aids only for outside activity; and (iii) walking without aids. No difference was made in the mobility score based on whether the walking aids were one or two crutches or a rollator. Patient reported outcome measures (PROM) were collected digitally preoperatively and at 3 and 12 months postoperatively, including a modified Harris hip score (HHS)^34^, the Oxford hip score (OHS)^35^, the hip disability and osteoarthritis outcome score (HOOS)^36^, the EQ‐5D‐3L^37^, and the visual analogue scale for pain (VAS). All complications were scored up to 1 year postoperatively, including dislocation, neuropathy, infection, thromboembolic events, and periprosthetic fractures.

### *Harris Hip Score*

A modification (i.e. without the physical examination section) of the HHS (mHHS) was used to evaluate postoperative recovery of hip function in an adult population^38^, ^39^. The mHHS system is based on pain and function of the patient. The scores could range from 0 (poor function) to a maximum of 100 points (excellent function).

### *Oxford Hip Score*

The OHS is a disease‐specific 12‐item questionnaire on the perception of patients with total hip replacement^35^. Each question contains five quantifiable answering possibilities, with a score of 0 for the worst and 4 for the best outcome, leading to a total score that can range between 0 (worst outcome) and 48 (best outcome).

### *Hip Disability and Osteoarthritis Outcome Score*

The HOOS includes five subscales: pain, other symptoms, function in daily living, function in sport and recreation, and hip‐related quality of life^36^, with a total of 40 questions. Standardized response options are given (five‐point Likert scale) and each question is scored from 0 to 4; a normalized score (100 indicating no symptoms and 0 indicating extreme symptoms) can then be calculated for each subscale.

### *EQ‐5D‐3L*

The EQ‐5D‐3L is a generic measure of health including five questions regarding mobility, self‐care, usual activities, pain/discomfort, and anxiety/depression^40^. Each question has three options for response: no problems (scored as 1), some problems (scored as 2), and extreme problems (scored as 3). The combination of these answers was subsequently converted to a single summary index score using population‐specific weights^37^. Scores then ranged from −0.329 (worst score) to 1. In addition, a VAS thermometer is included on which patients value their health state (0 = worst imaginable health state, 100 = best imaginable health state).

### *Visual Analogue Scale for Pain*

The VAS for pain at rest and the VAS for pain during activities was scored from 0 to 100, with 0 being no pain and 100 maximal pain.

### *Radiological Outcome*

Femoral and acetabular component positions were analyzed on standard anteroposterior pelvic radiographs performed 6 weeks postoperatively. Acetabular component inclination was measured by the angle between a horizontal pelvic line through the lower edge of the left and right obturator foramen and the major diameter of the ellipse represented by the rim of the acetabular cup. Leg length difference was determined by subtracting the vertical distance from the horizontal pelvic line to a point chosen on the lesser trochanter on the non‐operated leg from the distance from the horizontal pelvic line to the same point chosen on the trochanter minor of the operated leg. Femoral component position was measured by the angle of the femoral shaft axis and the long axis of the femoral stem. Femoral component sizing was scored for adequate femoral filling or undersizing. Subsidence, wear, and osteolysis were measured on a standard anteroposterior pelvic radiograph 1‐year postoperatively.

### *Statistical Analyses*

Patient characteristics and outcome parameters were considered to have a normal distribution based on histogram plots. Unpaired *t*‐tests were used to analyze differences between the DSA group and the MPA group in age, BMI, surgical time, perioperative blood loss, hemoglobin level change, length of hospital stay, acetabular inclination, femoral varus position, and leg length difference. χ^2^‐tests were performed to analyze differences between the groups in gender, primary diagnosis, smoking habit, acetabular Trident component, femoral component undersizing, discharge to home destination, and number of complications, and χ^2^‐tests for trend were used to analyze differences in ASA classification, Charnley score, and mobility score. Two‐way repeated measures ANOVA was used to analyze pain scores and PROM between the DSA and the MPA group. To analyze any learning curve in surgical time, as well as perioperative blood loss and changes in hemoglobin levels, the DSA group was subdivided into three subgroups based on the date of surgery: patients 1–17, 18–35, and 36–52. Analysis of the three subgroups was done by one‐way ANOVA. A *P*‐value below 0.05 was considered a significant difference. IBM SPSS Statistics version 24 was used for the statistical analyses.

## Results

### *Follow‐up*

The follow‐up period of this study was 1 year. A total of 3 patients were lost to follow up at 1 year: 1 in the DSA group and 2 in the MPA group, of which 1 had died because of non‐surgical‐related causes 6 months after THA.

### *Surgical Outcome*

Patient characteristics of the DSA and MPA groups were not different in age, gender, BMI, ASA classification, diagnosis, Charnley score, or smoking habit (Table 1). The surgical outcome parameters (Table 2) only revealed a significantly higher surgical time in the DSA (61 min, SD 8) compared to the MPA group (46 min, SD 12, *P* < 0.001). Within the DSA group, no measurable learning effect was observed in surgical time, perioperative blood loss, or hemoglobin level change after surgery (Table 3).

**Table 1:** Patient characteristics

	Direct superior approach, *N* = 52	Mini posterior approach, *N* = 52	*P*‐value
Age† (years)	69 (8.4)	69 (8.4)	0.63
Gender‡ (male)	24 (46%)	18 (35%)	0.23
Body mass index† (kg/m2)	25 (3.4)	25 (2.7)	0.65
ASA classification‡			
I	14 (27%)	13 (25%)	0.86
II	34 (65%)	35 (67%)	
III	4 (8%)	4 (8%)	
Diagnosis primary osteoarthritis‡	51 (98%)	50 (96%)	0.56
Charnley score‡			
A	21 (40%)	27 (52%)	0.14
B1	19 (37%)	16 (33%)	
B2	8 (15%)	9 (15%)	
C	4 (8%)	0 (0%)	
Smoking habit‡	5 (10%)	8 (15%)	0.37

† The values are given as mean with standard deviation.

‡ The values are given as number of patients with percentage. ASA, American Society of Anaesthesiologists.

**Table 2:** Results direct superior approach *vs* mini posterior approach

	Direct superior approach	Mini posterior approach	*P*‐value
Surgical time† (min)	61 (8)	46 (12)	<0.001
Blood loss perioperative† (mL)	313 (146)	302 (248)	0.80
Decrease in hemoglobin level† (mmol/L)	1.4 (0.4)	1.4 (0.5)	0.91
Pain score† (number)			
Postoperative	2.4 (1.5)	2.7 (2.1)	0.16
At discharge	2.0 (1.0)	2.3 (1.3)	
Length of stay† (days)	2.4 (1.2)	2.8 (1.1)	0.16
Discharge to home‡	48 (92%)	48 (92%)	1.00
Mobility at 6 weeks‡			
Aids for inside	3 (6%)	5 (10%)	0.12
Aids for outside	16 (31%)	22 (42%)	
Walking without aids	33 (63%)	25 (48%)	
Mobility at 1 year‡			
Aids for inside	1 (2%)	1 (2%)	0.36
Aids for outside	2 (4%)	0 (0%)	
Walking without aids	43 (94%)	45 (98%)	
Complications‡			
Total	2 (4%)	4 (8%)	0.40
Dislocation	0	2	
Periprosthetic fracture	2	1	
Ischial neuropathy	0	1	
Infection	0	0	
Thromboembolic event	0	0	
Acetabular component Trident‡	51 (98%)	48 (92%)	0.17
Acetabular component inclination† (degrees)	51 (6)	54 (7)	0.028
Femoral component varus† (degrees)	1.1 (1.8)	1.2 (1.3)	0.61
Femoral component undersizing‡	8 (15%)	9 (17%)	0.79
Leg length difference† (cm)	0.3 (0.5)	0.1 (0.5)	0.035

† The values are given as mean with standard deviation.

‡ The values are given as number of patients with percentage.

**Table 3:** Results for learning curve direct superior approach

	Direct superior approach			*P*‐value
	Patients 1–17	Patients 18–35	Patients 36–52	
Surgical time† (min)	59 (8)	65 (8)	59 (7)	0.07
Blood loss perioperatively† (mL)	319 (164)	347 (129)	269 (142)	0.29
Hemoglobin level change† (mmol/L)	1.4 (0.4)	1.5 (0.4)	1.3 (0.5)	0.62

† The values are given as mean with standard deviation

### *Clinical Outcome*

Repeated measures analysis showed no interaction between the mean pain score over time and the surgical approach (*P* = 0.86). In general, the mean pain scores decreased from a VAS score of 2.6 (SD 1.8) postoperatively to 2.1 (SD 1.2) at discharge from the hospital (*P* = 0.030), but there was no difference in mean pain scores between the DSA group and the MPA group (*P* = 0.16, Table 2). The mean length of stay in the hospital was comparable (*P* = 0.16), at 2.4 days (SD 1.2) in the DSA group and 2.8 days (SD 1.1) in the MPA group. In both groups, 92% of the patients were discharged to their home.

### *Radiological Outcome*

Acetabular component inclination was lower (*P* = 0.028, Table 2) in the DSA group (mean 51°, SD 6) compared to the MPA group (mean 54°, SD 7). The mean leg length difference postoperatively of 3 mm (SD 5 mm) in the DSA group was significantly larger (*P* = 0.035) compared to 1 mm (SD 5 mm) in the MPA group. No significant differences were found between the DSA and MPA groups in the percentage of Trident acetabular components used, the varus positioning of the femoral components, and the number of undersized femoral components. No subsidence, wear, or osteolysis was observed 1 year postoperatively.

### *Functional Outcome*

The mobility scores were not different at 6 weeks and 1 year after surgery (*P* = 0.12 and *P* = 0.36 respectively). As can be seen in Table 4, all PROM improved postoperatively in both the DSA and the MPA group (*P* < 0.01). There were no significant differences in PROM between the DSA and the MPA group (Table 4).

**Table 4:** Patient reported outcome measures

		Direct superior approach	Mini posterior approach	*P*‐value
VAS pain at rest	Preoperative	47 (23.2)	47 (24.0)	0.63	
	3 months postoperative	6 (8.9)	9 (15.2)	
VAS pain during activities	Preoperative	71 (19.8)	73 (20.3)	0.38	
	3 months postoperative	11 (21.4)	15 (21.6)	
Modified Harris hip score	Preoperative	56 (16.0)	54 (15.0)	0.75	
	3 months postoperative	87 (16.9)	85 (16.4)	
	1 year postoperative	87 (16.2)	89 (14.8)	
Oxford hip score	Preoperative	24 (5.7)	24 (8.8)	0.89	
	3 months postoperative	41 (7.1)	41 (6.5)	
	1 year postoperative	44 (6.3)	43 (6.0)	
HOOS pain	Preoperative	36 (8.0)	38 (19.0)	0.93	
	3 months postoperative	85 (14.4)	85 (17.0)	
	1 year postoperative	90 (13.0)	88 (17.9)	
HOOS symptoms	Preoperative	40 (13.3)	36 (17.4)	0.50	
	3 months postoperative	78 (14.7)	77 (16.0)
	1 year postoperative	84 (15.6)	85 (15.9)	
HOOS daily activities	Preoperative	36 (9.7)	35 (16.8)	0.86	
	3 months postoperative	82 (16.7)	82 (15.9)	
	1 year postoperative	87 (16.9)	86 (18.6)	
HOOS quality of life	Preoperative	23 (11.9)	21 (15.4)	0.93	
	3 months postoperative	71 (21.4)	70 (22.0)	
	1 year postoperative	76 (21.2)	79 (22.4)	
HOOS sport	Preoperative	14 (12.3)	16 (14.2)	0.75	
	3 months postoperative	69 (23.4)	70 (26.8)	
	1 year postoperative	76 (24.3)	77 (29.1)	
EQ‐5D total	Preoperative	0.54 (0.260)	0.53 (0.308)	0.69	
	3 months postoperative	0.87 (0.164)	0.85 (0.183)	
	1 year postoperative	0.89 (0.154)	0.88 (0.144)	
EQ‐5D VAS	Preoperative	62 (19.2)	66 (23.5)	0.64	
	3 months postoperative	80 (16.9)	80 (19.2)	

All values are given as mean with standard deviation

HOOS, hip disability and osteoarthritis outcome score; VAS, visual analogue scale.

### *Complications*

Complications included two patients with Vancouver B2 periprosthetic fractures in the DSA group. In one of these patients the fracture occurred after a traffic accident, 9 weeks postoperatively. At that time, the patient was pain free and fully functional without aids. The X‐ray at the patient’s regular visit before the accident showed no abnormalities. The other patient had a fracture without adequate trauma at 3 weeks follow up. Therefore, the fracture was regarded as surgery related. Both patients needed femoral stem revision. In the MPA group, 1 patient received a stem revision because of a Vancouver B2 periprosthetic fracture without an adequate trauma 4 weeks after the operation. In the MPA group, 2 patients had a dislocation of their hip prosthesis: 1 directly postoperatively who received an arthrotomy with revision to a longer femoral head, and the other 3 weeks postoperatively, who received a closed reduction. One ischial neuropathy occurred in the MPA group, which persisted for 1 year postoperatively and for which a walking aid was needed. No infections or thromboembolic events were observed. In total, 4% of complications were observed in the DSA group compared to 8% in the MPA group, which was not significantly different (*P* = 0.40).

## Discussion

### *Surgical, Clinical, and Functional Outcomes*

In this study, we presented the learning curve of the recently introduced the DSA technique for THA as experienced by two well‐trained, high volume orthopaedic hip surgeons. We compared the surgical, clinical, and radiological results with a matched control group of patients operated on using the MPA. No significant learning effect was found in the DSA group regarding surgical time, perioperative blood loss, and change in perioperative hemoglobin level. Compared to the MPA group, the mean surgical time was higher in the DSA group, but no significant differences were found in blood loss, length of stay in the hospital, discharge destination, mobility after 6 weeks and 1 year, complications, or PROM. As there is no learning effect using the DSA, excluding any patients from institutes using the DSA to prevent effects from a learning curve in future register studies is not required.

The mean surgical time, perioperative blood loss, and length of stay in the hospital in the DSA group of the current study was comparable to the pioneer study with the DSA^25^. Similar to Nam *et al*. we did not find significant differences in pain scores between DSA and MPA groups^7^. However, lower pain scores, decreased length of stay, and increased mobility after 6 weeks were observed in favor of the DSA group, although this was not statistically significant, possibly due to a lack of power. The fact that we did not find any differences in PROM between DSA and MPA groups after 3 months and 1 year might be the result of an expected beneficial effect only within the first 3 months. Capuano *et al*. found a faster return to normal activity and improvement in the postoperative Harris hip score up to 1 year in their tissue‐sparing posterior superior approach, which is synonymous to the DSA^26^.

### *Radiological Outcome*

Finally, we did not find any compromise in the acetabular or femoral component position. Although the difference in cup inclination and leg length was significant, we believe a difference of 3° inclination and 2‐mm leg length is not clinically relevant. Direct access to the acetabulum and femur can easily be obtained and the angulated reamer used in the DSA may even be favorable for the cup positioning. In the DSA, there is no direct visibility of the minor trochanter, which many surgeons use as a reference point for the osteotomy level of the femoral neck. The level of neck resection usually follows the line of the external obturator muscle from the piriform fossa but can be chosen too proximal in the DSA. The correct level of the osteotomy can be measured perioperatively from the tip or center of the femoral head or piriform fossa from the preoperative templating. In addition, a patient‐specific osteotomy guide can be used to perform the femoral neck osteotomy in the DSA^41^.

### *Complications*

With regard to the safety of the DSA, the two complications in the DSA group were both periprosthetic fractures: in 1 patient the fracture was due to high energy trauma and in 1 patient the fracture was surgery related. No dislocations, ischial neuropathy, infections, or thromboembolic events were observed in the DSA group. Supercapsular percutaneously‐assisted total hip replacement (SuperPATH) is also a micro posterior approach in which 9 periprosthetic fractures were found in a cohort study of 330 patients (3%)^42^. In addition, Roger and Hill reported a low incidence of complications (of 3) in 135 patients operated on using the DSA^25^. Tsiridis *et al*. (2019) reported two complications in 200 cases: one acute deep and one superficial wound infection^29^. In the DSA, only the piriformis and internal obturator tendon are released. The external rotator muscles distal to the internal obturator tendon are left in place. Bringing the leg up to 40 degrees of internal rotation rather than 90 degrees during the femoral preparation further decreases detachment from the distal external rotators without compromising visibility and direct access to the femur. Sparing the iliotibial band, the distal external rotators and superior capsulotomy with direct repair^26^ could lead to more hip stability and thereby explain that no dislocations were observed in the DSA patient group. In addition, Tsiridis *et al*. did not find any dislocations in 200 patients receiving THP using the DSA^29^.

### *Limitations and Future Prospects*

The major limitation of the current study is the lack of long‐term follow up. However, this study has shown an adequate acetabular and femoral component position and the 10‐year survival of the Trident cup and the Accolade TMZF stems already as high as 95% with an ODEP rating of 10A*^43^. Long‐term follow up after hip replacement with the DSA is needed to show that the proven durability of total hip replacement is not being compromised by an alternative exposure. The DSA is promising for the future as there is no learning curve, which could be related to the familiarity to the landmarks used by posterior‐oriented hip surgeons with direct access to both the acetabulum and the femur. The only other iliotibial tract‐sparing hip approach is the direct anterior approach (DAA). However, the DAA is more difficult to adopt for surgeons familiar with the posterior approach and can be associated with a 20% complication rate during the long learning curve^14^, ^15^, ^16^, ^17^, although some surgeons have shown that this transition can be made safely^19^. In both the DAA and the DSA, patients can benefit from the tissue‐sparing exposure^44^. However, the DSA is also promising as the DSA can easily be extended distally to a conventional posterior approach if needed.

## Conclusion

In conclusion, this is the first study demonstrating that hip replacement with the DSA can be introduced safely without a measurable learning curve and without an increase in complications. Only a slight increase in operation time is expected in the early experiences of using the procedure. In this study, we demonstrate that it is safe for surgeons who are familiar with the MPA to proceed with application of the DSA for THA.
